# Oral delivery of nerolidol alleviates cyclophosphamide-induced renal inflammation, apoptosis, and fibrosis via modulation of NF-κB/cleaved caspase-3/TGF-β signaling molecules

**DOI:** 10.1080/10717544.2023.2241661

**Published:** 2023-08-09

**Authors:** Ashif Iqubal, Abul Kalam Najmi, Shadab Md, Huda Mohammed Alkreathy, Javed Ali, Mansoor Ali Syed, Syed Ehtaishamul Haque

**Affiliations:** a Department of Pharmacology, School of Pharmaceutical Education and Research, New Delhi, India; b Department of Pharmaceutics, Faculty of Pharmacy, King Abdulaziz University, Jeddah, Saudi Arabia; c Department of Pharmacology, Faculty of Medicine, King Abdulaziz University, Jeddah, Saudi Arabia; d Department of Pharmaceutics, School of Pharmaceutical Education and Research, New Delhi, India; e Department of Biotechnology, Jamia Millia Islamia, New Delhi, India

**Keywords:** Nephrotoxicity, oxidative stress, renal fibrosis and inflammation

## Abstract

Cyclophosphamide (CP) is one of the most extensively used antineoplastic drug, but the nephrotoxicity caused by this drug is a major limiting factor for its use. Nerolidol (NERO) is a natural bioactive compound with diverse pharmacological actions. *In Vitro* and *in vivo study* was performed using HK-2 renal cells and Swiss Albino mice. Cell lines and animals were treated with NERO 25 and 50 µM + 30 µM CP (*in vitro*), 200 and 400 mg/kg, p.o. NERO from day 1 to day 15 + 200 mg/kg, i.p. CP on day 17 as single intraperitoneal injection (*in vivo*). The makers of oxidative stress, renal-specific injury markers, inflammation, apoptosis, fibrosis, and histopathological changes were studied. The study’s outcome showed a significant reduction in the level of malonaldehyde and interleukin-6 (p < 0.01), tumor necrosis factor-α, IL-1β (p < 0.001), and an increase in the superoxide dismutase, catalase, glutathione and interleukin-10 level (p < 0.01), in the *in vivo* study when treated with NERO 400 and compared with CP 200. *In Vitro* study showed reduced expression of nuclear factor kappa light chain enhancer of activated B cells, cleaved caspase-3, kidney injury molecule-1 and transforming growth factor-β-1 (p < 0.001), when treated with NERO 50 µM whereas NERO 25 µM only reduced the level of cleaved caspase-3 (p < 0.05) when compared with 30 µM. NERO 400 also reduced uric acid (p < 0.05), urea (p < 0.01), blood urea nitrogen, and serum creatinine levels (p < 0.001) and increased the level of blood-urea-nitrogen/creatinine ratio (p < 0.001). Additionally, the level of fibrosis-specific markers such as transforming growth factor-β1, hyaluronic acid (p < 0.01), 4-hydroxyproline, a collagen-rich area in Masson’s’ trichome stain, and Smad3 expression was also significantly reduced (p < 0.001). Furthermore, the outcome of multiple renal staining showed structural reversal aberrations, reduction of the thick basement membrane, and glycogen level toward normal when treated with NERO 400. Thus, the study showed a novel mechanistic modality of NERO against cyclophosphamide-induced renal toxicity. The outcome of this study can be considered a step closer to the development of an adjuvant to mitigate cyclophosphamide-induced renal toxicity among patients treated with cyclophosphamide.

## 1. Introduction

Cyclophosphamide CP) is among the most extensively and explicitly used anticancer drugs in various types of solid tumors and hematological malignancies. Apart from being a commonly used anticancer drug, it is also an immunosuppressive agent and uses in nephrotic syndrome (Hu et al., 2022). Pharmacokinetically, it is a prodrug and hence undergoes hepatic metabolism. In the liver, CP is acted upon by CYP34A and converted into phosphoramide mustard and acrolein. Phosphoramide mustard acts as an active metabolite and exhibits a potent anticancer effect via binding to N-7 of the guanine residue and altering DNA replication (Barnett et al., 2021). Acrolein, another active metabolite of CP, a small-length carbon chain and is hydrophobic in nature. Acrolein and other metabolites, such as hydroxyphosphamide, are reported to exhibit deleterious effects on various organs. Thus. When CP is administered for therapeutic purposes, it also exhibits significant toxicity, apart from its anticancer effect (Mills & Roberts, 1979). This often limit its widespread use and negatively affect patients’ quality of life. Among the various toxic manifestations of CP, nephrotoxicity is commonly observed at the therapeutic dose (Ayza et al., 2022). The kidney is an essential vital organ that regulates intracellular and extracellular physiological functions. In other words, the kidney maintains the ionic gradient, pH, fluidity, and overall body homeostasis. Thus, any alteration in kidney function directly affects the body’s overall function (Ayza et al., 2022).

Thus, understanding the molecular and cellular mechanisms of CP-induced renal toxicity is important. According to published evidence, persistent production of reactive oxygen species (ROS), reactive nitrogen species (RNS), decreased activity of antioxidant enzymes such as catalase (CAT), superoxide dismutase (SOD), an antioxidant such as glutathione (GSH), and an increase in the level of malondialdehyde (MDA) are significant (Mahipal & Pawar, 2017). Moreover, CP administration-induced oxidative stress also causes inflammation via modulation of the nuclear factor erythroid 2–related factor 2 (Nrf2), NLR family pyrin domain containing 3 (NLRP3), nuclear factor kappa-light-chain-enhancer of activated B cells (NF-κB), and p38 mitogen-activated protein kinase (MAPK) pathways (Lin et al., 2020). Moreover, the administration also causes apoptosis via increased activity of cytochrome C, mitochondrial dysfunction, increased expression, and levels of cleaved caspase-3 and other pro-apoptotic proteins (Zhang et al., 2021). It is also important to mention that we have previously established the pro-fibrotic effect of CP in cardiac and liver tissue (Iqubal et al., 2019). Thus, here also, we explored the same pattern of toxicity in renal tissue. Because the CP also produces transforming growth factor beta (TGF-β), it contributes to the fibrotic cascade alongside collagen production. Hence, CP administration causes persistent apoptosis, oxidative stress, inflammation, and fibrosis, leading to multifactorial nephrotoxic manifestations.

Thus, an unmet need exists for an adjuvant to mitigate CP-induced renal toxicity. Recently, natural products have been extensively studied for diverse pharmacological effects (Ayza et al., 2022). Hence, in the present study, we have also explored the renal protective effect of a monocyclic sesquiterpene, Nerolidol (NERO). NERO belongs to the class of secondary metabolites and possesses multiple health benefits (Chan et al., 2016) The FDA approves it for use as a flavoring agent and as a food additive (Chan et al., 2016) It is also categorized as GRAS; hence, its safety is also assured. Previous studies have shown its potent antioxidant, anti-inflammatory, anti-apoptotic, neuroprotective, hepatoprotective, neuroprotective, and anticancer effects (Chan et al., 2016). However, until now, its effect on cyclophosphamide-induced nephrotoxic manifestations (oxidative stress, inflammation, apoptosis, and fibrosis) has not been studied.

This current study investigated nerolidol’s renal protective potency against cyclophosphamide-induced renal oxidative stress, inflammation, apoptosis, and fibrosis in an *in vitro* and *in vivo* model via the NF-κB, caspase-3, TGF-β/Smad3 axis. Moreover, to validate our finding, we also explored the pro-fibrotic effect of cyclophosphamide. We also explored the histopathological aberration, along with periodic acid–Schiff (PAS), Jones methenamine silver (JMS), and Masson’s Trichrome (MT) staining.

## 2. Materials and methods

For the induction of renal toxicity in the *in vivo* and *in vitro* models, cyclophosphamide and acrolein were procured from Sigma Aldrich, USA. Nerolidol used in the study was also procured from Sigma Aldrich, USA. ELISA kits used to estimate inflammatory and fibrotic markers were procured from Krishgen Biosystem, Mumbai. The antibody used for immunohistochemistry was procured from Santa Cruz Biotechnology. All other chemicals and reagents used in the experiment were of analytical grade.

### 2.1. In vitro study

For the *in vitro* study, human renal tubular HK-2 cells (CRL-2190, ATCC) were used. The cells were incubated in a DMEM/F12 medium that contained fetal bovine serum (10%), 100 mg/ml streptomycin, and 100 μ/L of penicillin at 37 °C in 5% CO_2_ (Raj et al., 2020). To assess the expression of various markers, 5 × 10^3^cell density was used, and 30 μM of the cyclophosphamide metabolite (acrolein) was added into a 6-well plate along with 25 and 50 µM of nerolidol and left for 24 h. After 24 h, cells were obtained for assessing various inflammatory, apoptotic, and fibrotic markers (Raj et al., 2020).

#### 2.1.1. Estimation of gene expression of NF-κB, caspase-3, TGF-β and KIM-1 using RT-PCR

NF-κB, caspase-3, KIM-1, and TGF-β expression was studied using RT-PCR. For this assay, drug-treated HK-2 was used to isolate total RNA using an RNase commercial kit obtained from Qiagen and processed as per the manufacturer’s instruction. NanoDrop 1000™ obtained from Thermo Scientific was used for the determination of quality as well as quantity of RNA. The obtained 1 μg of RNA was used for the cDNA preparation using reverse transcription reaction obtained from Bio-Rad. The sequence of a primer used in this RT-PCR is shown in Table 1. The efficacy of the primer was evaluated by the dilution and the gradient temperature technique (50–55). The PCR process was conducted using 20 μl of the prepared sample having one μl cDNA, 500 ng/μl final concentration of primer, and EvaGreen supermix qPCR obtained from Biorad. The 2^-ΔΔCt^ method was used for the estimation of fold change, and the experiment was conducted in triplicate (Heeba & Mahmoud, 2016).

**Table 1.** Primers used in the study.

Gene	Forward primers	Reverse primers	Accession No.
NF-κB	CTGGTGGACACATACAGGAAGAC	ATAGGCACTGTCTTCTTTCACCTC	NM_019408
Caspase-3	ATCCATGGAAGCAAGTCGAT	CCTTTTGCTGTGATCTTCCT	NM_012922.2
KIM-1	TGGCACTGTGACATCCTCAGA	GCAACGGACATGCCAACATA	NM_173149
TGF-β1	CCCTACATTTGGAGCCTGGA	TAGTAGACGATGGGCAGTGG	NM_03104
GAPDH	GTTACCAGGGCTGCCTTCTC	GATGGTGATGGGTTTCCCGT	NM_017008

### 2.2. Experimental animals in vivo study

Swiss Albino mice (30–35 grams) were used in the *in vivo* study. Experimental animals were provided by the central animal house facility of Jamia Hamdard (IAEC/JH/1484) after approval from the Committee for Control and Supervision of Experiments on Animals (CPSEA). The animals were acclimatized for one week before the initiation of the experiment and kept on the standard diet, temperature, and humidity in a propylene cage.

### 2.3. Treatment regimen for the in vivo study

**Figure 1.**
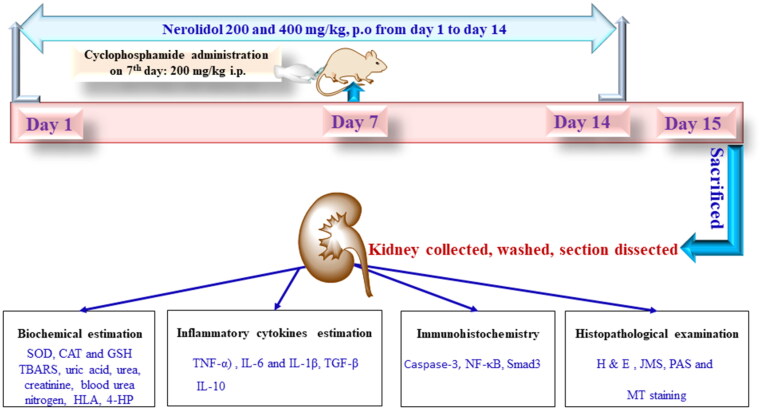
Shows the treatment plan for the *in vivo* study. SOD; superoxide dismutase; CAT; catalase; GSH; glutathione; TBARS; thiobarbituric acid-reactive substances; HLA; hyaluronic acid; 4-hydroxyproline; TNF-α; tumour necrosis factor α, ILs; interleukins; TGF-β; transforming growth factor beta; NF-κB; nuclear factor kappa-light-chain enhancer of activated B cells, H&E; hematoxylin and eosin, JMS; jones methenamine silver, PAS; periodic acid–schiff, MT; masson’s trichrome.

For the *in vivo* study, 24 Swiss Albino mice were randomly divided into four groups (*n* = 6). Control (received normal saline from day 1 to day 14), CP 200 (received single injection of cyclophosphamide 200 mg/kg i.p. on day 7) CP 200 + NERO 200 (received nerolidol 200 mg/kg, p.o from day 1 to day 14 along with a single injection of cyclophosphamide 200 mg/kg i.p. on day 7) and CP 200 + NERO 400 (received nerolidol 400 mg/kg, p.o from day 1 to day 14 along with single injection of cyclophosphamide 200 mg/kg i.p. on day 7), as shown in Figure 1 (Iqubal et al., 2019). On day 15, animals were sacrificed, and blood was collected and stored for biochemical estimation. The kidney from all the animals was collected, rinsed with normal saline, weighed, and a section was cut and stored in 10% formalin for histopathological analysis. The remaining section was stored at −80 °C for biochemical analysis (Ghareeb et al., 2019).

#### 2.3.1. Estimation of the marker of oxidative stress

##### 2.3.1.1. Assessment of superoxide dismutase activity

Marklund & Marklund (1974) method was used to estimate SOD activity (Marklund & Marklund, 1974). Estimation of SOD was based on the principle that pyrogallol gets oxidized in the aqueous solution and at higher pH. Because of the chemical reactions, the solution turns yellowish-brown and green within a few, min and the color is produced, which further changes into yellow. Thus, in the assessment of SOD activity, superoxide anion radicals are responsible for the inhibition of pyrogallol’s auto-oxidation, and absorbance is recorded after the 10-second induction period. One unit (U) of the activity of SOD is the SOD enzyme that abrogated the pyrogallol’s auto-oxidation by 50%/min. For the estimation of SOD activity, 10% w/v tissue homogenate was used, i.e., 50 mg tissue was used and dissolved in 0.5 ml (500 µl) of phosphate buffer and further centrifuged at 10000 rpm for 20 min at 4 °C. From the supernatant, 100 µl was removed and added to 8.5 pH Tris HCL buffer. After adding Tris HCl buffer, 25 µl of pyrogallol (24 mM) was added, and the final volume (3 ml) was adjusted using the phosphate buffer and the solution and mixed. Absorbance was recorded at 420 nm for 3 min at the interval of 1 min, and the values were expressed as U/mg of protein/min (Marklund & Marklund, 1974).

##### 2.3.1.2. Assessment of catalase activity

Claiborne & Fridovich (1979) method was used for the estimation of CAT activity. Estimation was based on the principlRe thazat continuously increased absorption by reducing the wavelength is seen by H_2_O_2_ in the UV range. The reduced absorbance assesses the rate of H2O2 H_2_O_2_ decomposition at 240 nm, and the difference in absorption/time is considered as the activity of catalase. To estimate CAT activity, 10% w/v tissue homogenate was used, i.e., 50 mg tissue was used and dissolved in 0.5 ml (500 µl) of 50 µM/L phosphate buffer and centrifuged at 10000 rpm for 20 min at 4 °C. 50 µl of obtained supernatant was transferred to the cuvette that contains 2.95 ml of H_2_O_2_ (19 µM/L) that was also prepared in the potassium phosphate buffer. Absorbance was recorded at 240 nm for 3 min at the interval of 1 min, and the values were expressed as nanomoles of H_2_O_2_ per min per mg of protein (Claiborne & Fridovich, 1979).

##### 2.3.1.3. Assessment of glutathione level

Sedlak & Lindsay (1968) method was used for the GSH estimation (Sedlak & Lindsay, 1968). The estimation was based on the spectrophotometric principle that the –SH group causes a reduction of 5′ –dithiobis-2-nitrobenzoic acid (DNTB), leading to the formation of 2-nitro-5-mercaptobenzoic acid and exhibit yellow color and ultimately absorbance is recorded. For the assessment of GSH level, 150 mg of tissue was dissolved in 1.5 ml of 0.02 M EDTA and centrifuged at 10000 rpm for 20 min at 4 °C. EDTA (0.02 M) was prepared by the dissolving 22.3 g of EDTA in 300 ml of distilled water and 20 ml of the formed solution was further added to 200 ml of distilled water. 1 ml of obtained homogenate was then mixed with the distilled water (4 ml) and trichloroacetic acid (50%) and shaken for 10 min. After 10 min, the solution was centrifuged at 3000 rpm for 15 min, and 2 ml of supernatant was added to 4 ml Tris buffer (0.4 ml, pH 8.9) and 0.1 ml DTNB (0.01 M). Tris buffer was prepared by dissolving 24.2 g of Tris buffer in 100 ml of water, EDTA (0.2 M) was added to this solution, and the volume of 1 liter was adjusted, and the 8.9 pH was adjusted by using 1 N HCl. DTNB was prepared by dissolving (99 mg) to 25 ml Absolut methanol. The absorbance of the resultant mixture was recorded at 412 nm with 5 min of addition of DTNB, and values were expressed as µmole/mg of protein (Sedlak & Lindsay, 1968).

##### 2.3.1.4. Assessment of lipid peroxidation

Ohkawa et al. (1979) method was used to assess the MDA level (Ohkawa et al., 1979). The estimation was based on the principle that free radical’s reaction causes lipid peroxidation and produces peroxides that further get converted into MDA. Thus, the reaction of thiobarbituric acid along with MDA provides the amount of thiobarbituric acid reactive substances (TBARS); hence, the test is also called the TBARS test. To assess MDA level, 150 mg of tissue was dissolved in 1.5 ml of KCl (0.15 M) and centrifuged at 10000 rpm for 15 min at 4 °C). 0.15 M KCl was prepared by dissolving 2.3 g KCL to the 200 ml distilled water. 1 ml of suspension medium was taken after centrifugation, and TCA (30%) and TBA (0.8%) reagents, 0.5 ml each, were dissolved into it. Aluminum foil was used to cover the tube and shaken for 30 min at 80 °C. After this, the tubes were kept for 30 min in ice-cold water. After 30 min, tubes were centrifuged for 15 min at 3000 rpm, the absorbance was recorded at 540 nm, and the values were expressed as nanomoles of MDA per mg protein (Ohkawa et al., 1979).

#### 2.3.2. Estimation of renal injury markers

Renal injury markers such as creatine, uric acid, urea, albumin, and blood urea nitrogen (BUN) were estimated in serum using an autoanalyzer and as per the manufacturer’s instruction.

#### 2.3.3. Estimation of the marker of inflammation and fibrosis

Estimation of inflammatory cytokines and fibrotic markers such as IL-1β, IL-6, TNF-α, IL-10, TGF-β, HLA, and 4-HP was performed using the obtained and properly homogenized kidney sample as per the instruction provided in the commercially available ELISA kit from the manufacturer. For ELISA, the tissue homogenate was prepared using PBS buffer (pH 7.4) centrifuged at 4 °C using a glass homogenizer at 3000 rpm for 20 min, and the supernatant was collected for analysis. Sandwich ELISA kits for the markers mentioned above were stored at −80 °C. The standard was prepared by diluting the standard stock solution in the assay buffer. The plate was washed using the wash buffer, and any residual buffer was blotted by tapping the plates firmly on the absorbent paper. Firstly, standards and samples were added to the respective well along with the biotinylated antibody (except to the standard wells) and streptavidin:HRP Conjugate incubated and incubated for 60 min at 37 °C, and then the plate was washed using a wash buffer. In the following step, TMB Substrate was added to each well and incubated at 37 °C for 10 min. After 10 min, the blue color appeared, and Stop Solution was added to each well, and the wells were observed for color development of yellow color. Once the yellow color developed, absorbance was recorded within 10 -15 min of the Stop Solution at 450 nm (Khan et al., 2018)

#### 2.3.4. Histopathological (H & E, JMS, PAS, and MT staining) and immunohistochemical analysis

For the histopathological analysis (H & E, PAS, MT, and MS staining), stored tissue in 10% formalin was used. Samples were grossed and embedded in the paraffin block, and thin sections (5 µm) were cut using a microtome. Paraffinized slides were deparaffinized by keeping the slides on the warm table and kept in Xylene for 2-3 min, and the process was repeated 2-3 times. The slides were hydrated by passing in the graded alcohol in the decreasing order of 100-80-70-50 and 30% ethanol. Slides were kept in different concentrations for 1-2 min and washed with distilled water. In the next step, slides were kept in a Coplin jar for 3-5 min filled with Hematoxylin stain and washed with tap water. The next step is differentiation, where the excess dye was removed 0.5% HCl for a few seconds until nuclei appeared reddish or purple, and the slides were rinsed using tap water. The next step was bluing, where slides were kept in ammonia water for a few seconds, and the sections turned blue. Dehydration was done in ascending order by using 50% ethanol to 95% ethanol and counterstained in 1% Eosin for 1 min, washed in tap water for 1-3 min, and washed using absolute alcohol. In the last step, slides were kept in Xylene for 1 min for clearing and mounted in DPX (Ansari et al., 2019).

PAS staining was also conducted using the paraffinized blocks, and 5 µm thin sections were cut. PAS staining was done to identify glycogen/glycogen compounds. In this process, periodic acid oxidizes the tissue sections, resulting in aldehyde grouping and identifying using the Schiff reagent. Firstly, paraffin was removed from the slides and kept in 0.5% of periodic acid for 3-5 min for oxidation, and the slides were rinsed with distilled water. In the next step, slides were covered with Schiff reagent for 10-15 min, slides appeared light pink and washed for 5 min in lukewarm water, and the slides appeared dark pink. Further, the slides were counter-stained using Mayer’s Hematoxylin for 1-2 min and again washed with distilled water for 3-5 min. In the last step, slides were dehydrated and mounted in DPX (Jiang et al., 2020).

For the JMS staining also, paraffinized blocks and a 5 µm thin section were cut. MS staining is used to analyze the basement and glomerular basement membranes (GBM) in membranous nephropathy and nephrological disorder. In the first step, slides were deparaffinized, and 0.5% periodic acid was used for 10-15 min at 37 °C to oxidize tissue and rinsed with the distilled water. Slides were incubated in methenamine sliver for approximately 60 min at 60 °C and rinsed in warm water. In the next step, gold chloride solution was used for toning for 60 min and washed with distilled water. In the subsequent step, sodium thiosulfate was used for 120 min and washed in tap water for 10 min, followed by counterstaining in nuclear fast red or green (light) for 3-5 min. Finally, dehydration was done in Xylene and mounted in DPX (Hayashi et al., 1989).

For the MT staining, tissues were deparaffinized and further rehydrated using 100%-70% alcohol and washed in distilled water. Tissues were stained in Weigert’s iron hematoxylin solution for 7-10 min and washed with lukewarm water. In the next step, Biebrich scarlet-acid fuchsin stain was used for approximately 10 min and again washed with distilled water. Furthermore, tissues were differentiated in phosphomolybdic and phosphotungstic acid until the collagen appeared red. Once the red is visible, tissue is exposed to aniline solution for 10 min and differentiated in 1% acetic acid, immediately dehydrated using 95% alcohol to remove Biebrich scarlet-acid fuchsin stain. Finally, dehydration was done in Xylene and mounted in DPX (Albino et al., 2021).

For the immunohistochemical analysis, paraffinized blocks were cut into thin sections. Deparaffinized, washed, and rehydrated, followed by antigen and microwave retrieval. In the next step of the primary antibody reaction, slides were first incubated with 5% BSA for 7-10 min. The diluted antibody was spread over the slides, incubated for 1 hr. at 37 °C and washed with phosphate buffer. In the next step, a secondary antibody reaction takes place where the biotinylated secondary antibody is applied over slides, incubated for 0.5 hr (in the humified chamber), and washed with phosphate buffer. In the next step, the substrate mixture was applied to the slides, incubated for 7-10 min, and observed under the microscope. Finally, counterstaining was done using Mayer’s hematoxylin, incubated for approximately 5 min, washed under tap water, and slides were mounted (Althunibat et al., 2022). The image was captured using a Motic Inverted microscope and quantified using J image software (Khan et al., 2018)

### 2.4. Statical analysis

In the present study, values were stated as mean ± SEM. Statical analyses were performed using One-Way ANOVA followed by Tucky’s multiple comparison test with the help of Graph Pad software USA (version 6). P-value < 0.05 was considered statistically significant.

## 3. Result

### 3.1. Effect of nerolidol against CP-induced renal oxidative stress

**Figure 2.**
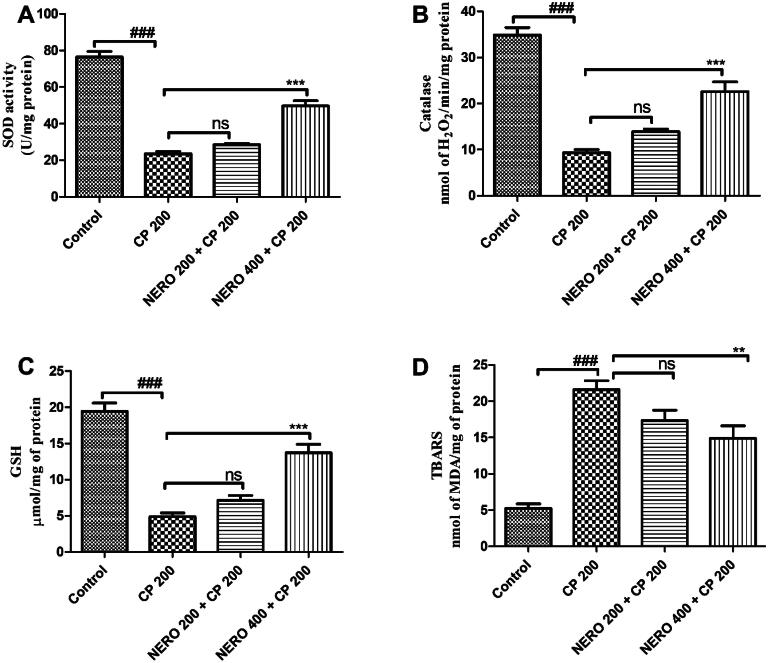
Showing the effect of nerolidol 200 and 400 mg/kg, p.o against cyclophosphamide-induced oxidative stress markers in the renal tissue. One-way ANOVA i.e., tukey’s multiple comparison tests was used for statistical analysis.

In the present study, we evaluate the renal protective effect of nerolidol against cyclophosphamide-induced renal toxicity. Based on the outcome of our study, we found that exposure to CP significantly increased the level of MDA and reduced the antioxidant activity of SOD, CAT, and level GSH (*P* < 0.001). Upon treatment with NERO 400 and NERO 200, NERO 400 significantly increased the antioxidant activity of SOD (*P* < 0.001), CAT (*P* < 0.001), GSH level (*P* < 0.001), and reduced the level of MDA toward normal (*P* < 0.01). However, treatment with NERO 200 did not exhibit a marked antioxidant effect against the derailed level of these oxidative stress markers (*P* > 0.05), as shown in Figure 2.

### 3.2. Effect of nerolidol against cyclophosphamide-induced renal inflammatory markers (NF-κB, TNF-α, IL-6, IL-1β, and IL-10)

**Figure 3.**
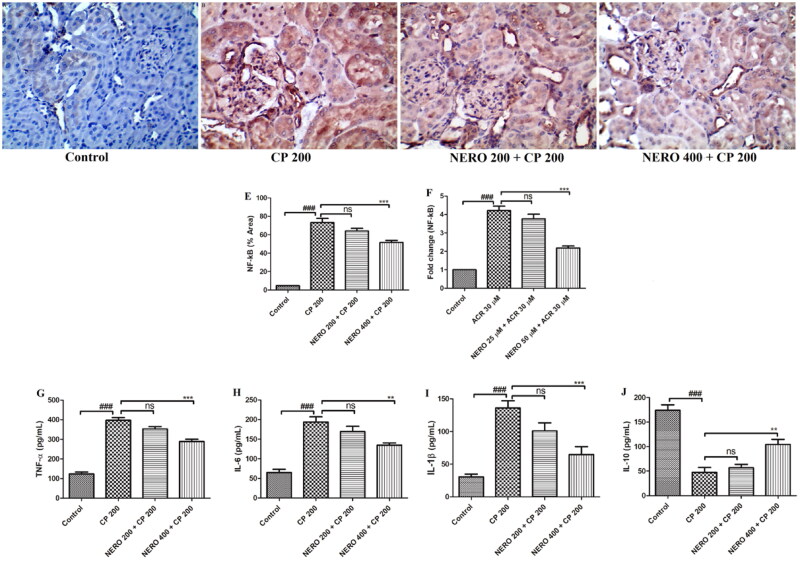
(A-D) Shows the effect of nerolidol 200 and 400 mg/kg, p.o against cyclophosphamide-induced markers of inflammation (NF-κB) in the renal tissue and figure E represents the semi-quantitative analysis of NF-κB level, figures (G-J) represent the effect of nerolidol 200 and 400 mg/kg, p.o against cyclophosphamide-induced markers of inflammation such as TNF-α, IK-6, Il-1β and IL-10, estimated in the *in vivo* study. (400 × magnification). figure F represents the expression of NF-κB from the *in vitro* study. One-way ANOVA i.e., tukey’s multiple comparison test was used for statistical analysis.

The in vitro study showed a significant increase in NF-(B mRNA expression when CP was exposed to HK-2 cells (*P* < 0.001). We also validated the finding of our *in vitro* study by performing immunohistochemistry of NF-κB in the *in vivo* study (renal tissue) and found a significant increase in the expression of NF-κB (*P* < 0.001). Moreover, our finding showed an increased level of IL-6 (*P* < 0.001), TNF-α (*P* < 0.001), IL-1β (*P* < 0.001), and a reduced level of IL-10 (*P* < 0.001) when compared to the control. When we tested the anti-inflammatory effect of NERO, we found that NERO, in the *in vitro* study, effectively reduced the expression of NF-κB (*P* < 0.001). In the *in vivo* study also, we found a marked reduction in the expression of NF-κB along with the decrease in the level of TNF-α (*P* < 0.001), IL-1β (*P* < 0.001), IL-6 (*P* < 0.01) as well as increased in the level of IL-10 (*P* > 0.01) as shown in Figure 3.

### 3.3. Effect of nerolidol against cyclophosphamide-induced renal apoptosis

**Figure 4.**
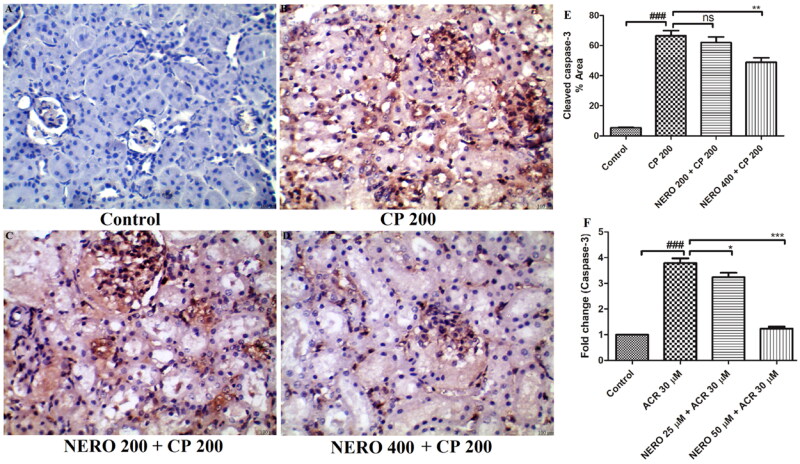
(A-D) Showing the effect of nerolidol 200 and 400 mg/kg, p.o against cyclophosphamide-induced markers of apoptosis (cleaved caspase-3) in the renal tissue, whereas figure E represents the semi-quantitative analysis of cleaved caspase level estimated in the *in vivo* study. Figure F represents the expression of caspase-3 from the *in vitro* study. One-way ANOVA i.e., tukey’s multiple comparison test was used for statistical analysis. (400 × magnification).

The findings of *in vitro* study showed increased expression of caspase-3 in the HK-2 cells when exposed to CP (*P* < 0.001). In the *in vivo* study, the outcome of immunohistochemical analysis also showed increased expression of cleaved caspase-3). When we tested the anti-apoptotic effect of NERO, we found that in the *in vitro* study, NERO effectively reduced the expression of caspase-3 (*P* < 0.001). In the *in vivo* study also, we found a marked reduction in the expression of cleaved caspase-3 (*P* > 0.001), as shown in Figure 4.

### 3.4. Effect of nerolidol against cyclophosphamide-induced renal injury markers

**Figure 5.**
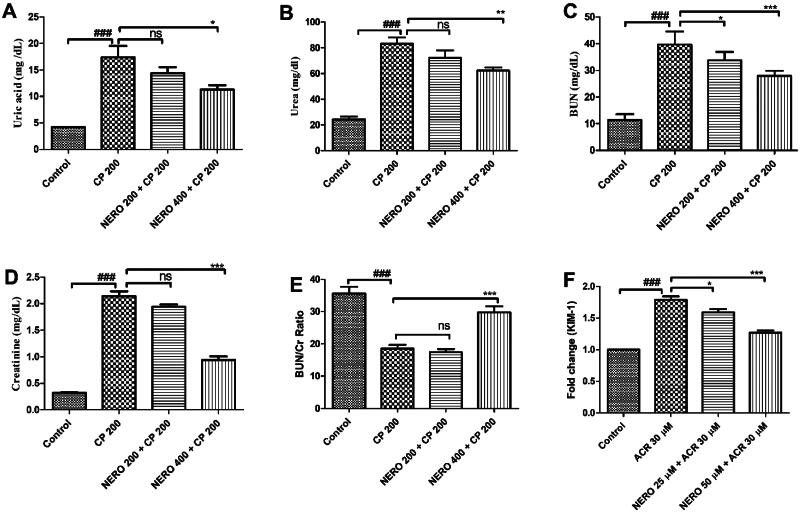
(A-E) Showing the effect of nerolidol 200 and 400 mg/kg, p.o against cyclophosphamide-induced markers of renal injury in serum such as uric acid, urea, blood urea nitrogen-creatinine ratio (BUN: Cr ratio), creatinine, and BUN in the renal tissue. Figure F represents the kidney injury molecule-1 (KIM-1) expression in the *in vitro* study. One-way ANOVA i.e., tukey’s multiple comparison test was used for statistical analysis.

In the present study, we found that CP 200 administration significantly increased the serum level of uric acid, urea, BUN, Cr, and BUN/Cr ratio, along with the expression of KIM-1. Treatment with NERO 400 significantly reversed UA (*P* < 0.05), urea (*P* < 0.01), BUN (*P* < 0.001), Cr (*P* < 0.001) level, and BUN/Cr ratio (*P* < 0.001) along with KIM-1 expression (*P* < 0.001) toward normal and hence signifies the nephroprotective effect. NERO 200 showed no significant nephroprotective effect (*P* > 0.05) as shown in Figure 5.

### 3.5. Effect of nerolidol against cyclophosphamide-induced renal histopathology (H &E staining)

**Figure 6.**
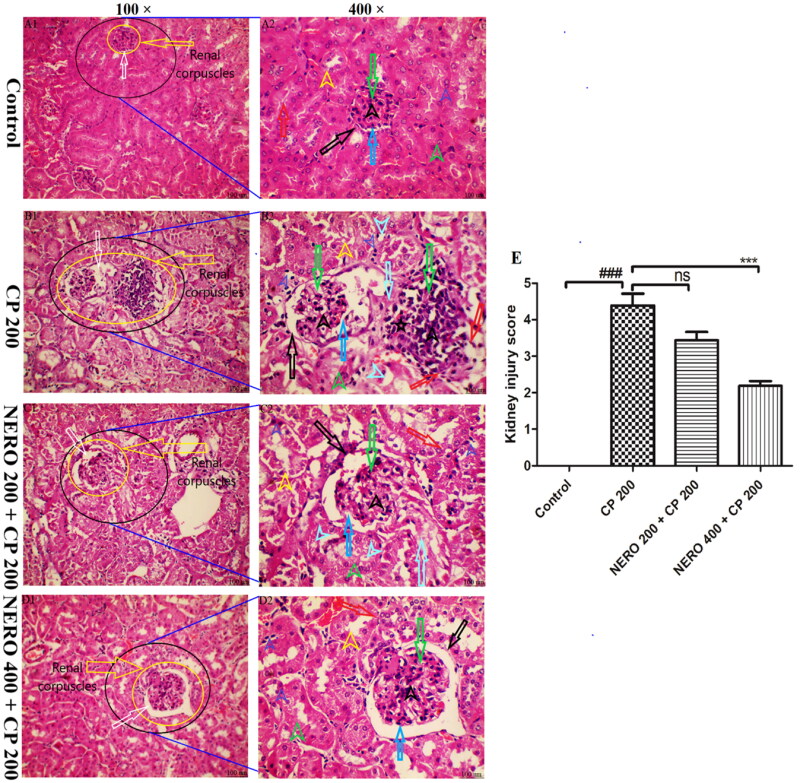
(A1-D1 and A2-D2) Showing the effect of nerolidol 200 and 400 mg/kg, p.o., against cyclophosphamide-induced histopathological aberrations (H & E staining) in the renal tissue. A1 -D1 showing renal corpuscles (glomerulus, bowman’s capsule, and bowman’s space) [yellow arrow] and bowman’s space [white arrow]. A2 -D2 showing glomerulus (long green arrow), parietal layer (long black arrow), cellular disintegration (long red arrow), podocytes (dark long blue arrow), fibrotic changes (long light blue arrow), mesangium with atrophic changes (short black arrow), pyknosis (short dark blue arrow), vacuolation (short light blue arrow), DCT (short yellow arrow) and PCT(short green arrow). figure E represents the semi-quantitative analysis of the kidney injury score. One-way ANOVA, i.e., tukey’s multiple comparison test was used for statistical analysis. (H & E staining, 100 × and 400 × magnification, respectively.).

The outcome of the present study showed a normal histological appearance of the renal section. Renal corpuscles (long yellow arrow), with the normal structure of glomerulus (long green arrow) and normal Bowman’s space (long white arrow), appear normal. Additionally, the histopathological analysis showed no sign of a damaged parietal layer (long black arrow), no cellular disintegration (long red arrow), no damaged podocytes (dark long blue arrow), no damaged mesangium (short black arrow), pyknosis (short dark blue arrow), vacuolation (short light blue arrow). Moreover, there was no damage to DCT and PCT (short yellow and green arrow) seen. When the animals were treated with 200 mg/kg cyclophosphamide, i.p as a single i.p injection on day 7, significant damage to renal corpuscles (long yellow arrow) and widening of Bowman’s space was found long white arrow). Additionally, significant damaged and disintegrated glomerulus, mesangium (long green and short black arrow), along with atrophic podocytes (dark long blue arrow) and damaged parietal layer (long black arrow) was seen. Moreover, the finding of the histopathological analysis showed a marked cellular disintegration (long red arrow), fibrotic changes (long light blue arrow), pyknosis (short dark blue arrow), vacuolation (short light blue arrow) along with damaged and disintegrated DCT and PCT (short yellow and green arrow) (*P* < 0.001). However, when NERO 200 and NERO 400 were used as adjuvants, the study’s outcome showed a significant reversal to aforementioned renal histopathological aberrations toward normal (*P* < 0.001) with NERO 400. In contrast, NERO 200 was ineffective against CP 200-induced damage, as shown in Figure 6.

### 3.6. Effect of nerolidol against cyclophosphamide-induced renal histopathology (JMS staining)

**Figure 7.**
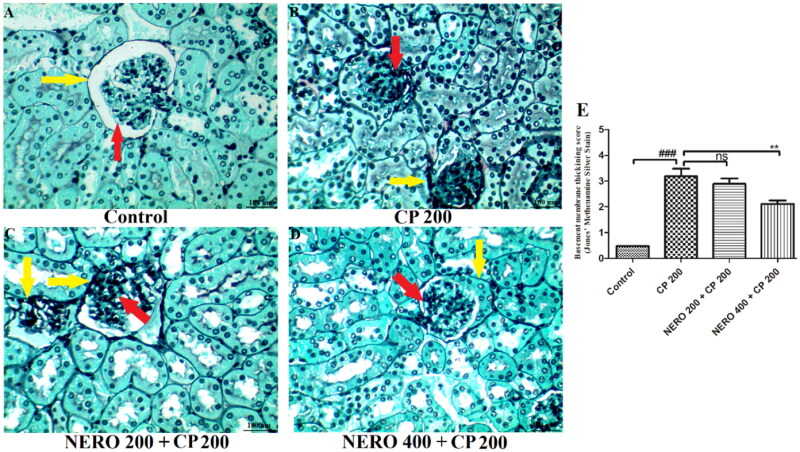
Showing the effect of nerolidol 200 and 400 mg/kg, p.o against cyclophosphamide-induced histopathological aberrations (JMS staining) in the renal tissue and figure E represents the semi-quantitative analysis basement membrane thickening score. One-way ANOVA i.e., tukey’s multiple comparison test was used for statistical analysis. (JMS staining, 400 × magnification).

In the present study, we found thickened and damaged glomerular and mesenchymal basement membranes upon CP 200 administration when compared with control groups (*P* < 0.001). When NERO 400 was used as an adjuvant, a significant reveal of these manifestations toward normal was found (*P* < 0.001), whereas NERO 200 was found to be ineffective (*P* > 0.05), as shown in Figure 7.

### 3.7. Effect of nerolidol against cyclophosphamide-induced renal histopathology (PAS staining)

**Figure 8.**
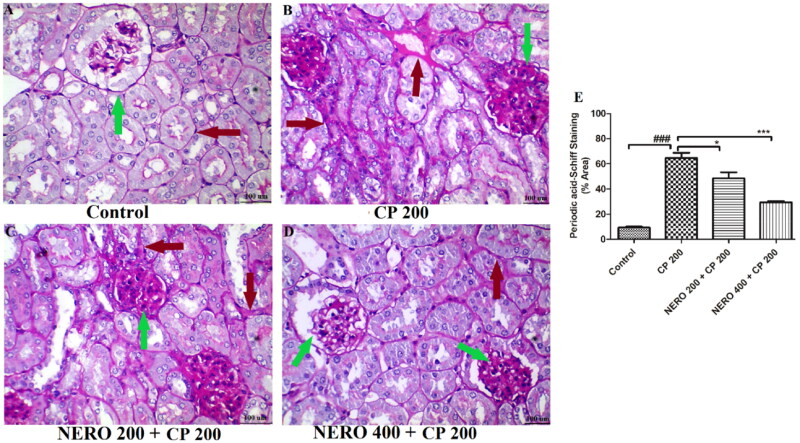
Showing the effect of nerolidol 200 and 400 mg/kg, p.o against cyclophosphamide-induced histopathological aberrations (PAS staining) in the renal tissue, and figure E represents the semi-quantitative analysis of PAS staining. One-way ANOVA, i.e., tukey’s multiple comparison test was used for statistical analysis. (PAS staining, 400 × magnification).

The present study’s finding showed the thickening of the glomerular and mesenchymal basement membrane and glycogen deposition in the glomerulus and the interstitials upon CP 200 administration. When NERO 400 was used as an adjuvant, a significant reversal of these manifestations toward normal was found (*P* < 0.001), and the findings of our study were in agreement with previously published reports, as mentioned above, whereas NERO 200 was found to be only mild effective (*P* < 0.05), as and shown in Figure 8.

### 3.8. Effect of nerolidol against cyclophosphamide-induced renal histopathology renal fibrosis and MT staining

**Figure 9.**
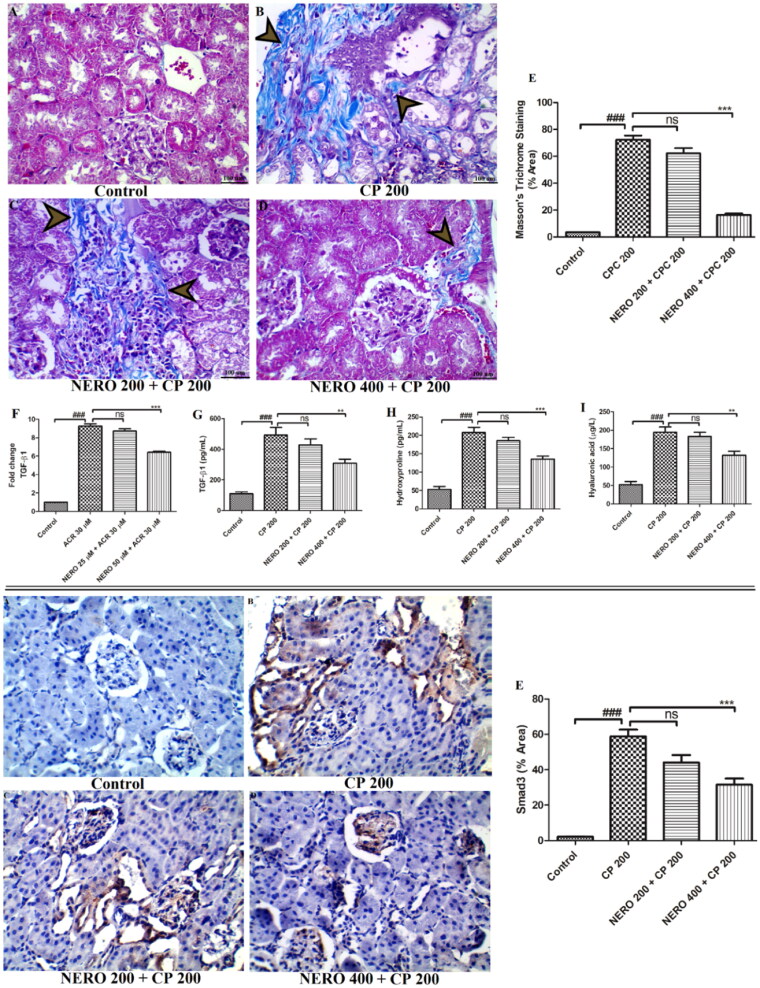
The upper panel (A-D) shows the effect of nerolidol 200 and 400 mg/kg, p.o against cyclophosphamide-induced renal fibrosis (MT staining, 400 × magnification), whereas figure E represents the semi-quantitative analysis of MT staining. Figure (F-I) represents the effect of nerolidol 200 and 400 mg/kg, p.o against cyclophosphamide-induced markers of renal fibrosis such as TNF-α, IL-6, IL-1β, and IL-10, estimated in the *in vivo* study. Lower panel (A-D) represents the effect of nerolidol 200 and 400 mg/kg, p.o against cyclophosphamide-induced Smad3 expression in the renal tissue (400 × magnification). figure E represents the semi-quantitative analysis of Smad3 level in renal tissue. One-way ANOVA, i.e., tukey’s multiple comparison test was used for statistical analysis.

In the present study, we have estimated the markers of renal fibrosis. We found that CP 200 increased the expression of TGF-β1 (*P* < 0.001), level of TGF-β1 (*P* < 0.001), level of HLA (*P* < 0.001), 4-HP (*P* < 0.001) and also increased the fibrotic area as evident from MT stain (*P* < 0.001). When we used NERO 200 and NERO 200, NERO 400 showed a marked reduction in the expression of TGF-β1 (*P* < 0.001), level of Smad3 (*P* < 0.001), HLA (*P* < 0.001), 4-HP (*P* < 0.01) and also reduced the collagen-rich area in the MT staining. Treatment with NERO 200, however, showed no significant antifibrotic effect (*P* > 0.05), as shown in Figure 9.

## 4. Discussion

The nephrotoxic manifestations of cyclophosphamide are well-established in clinical and preclinical studies (Ayza et al., 2022). However, the precise mechanism of nephron toxicity is not clear until now. Additionally, no adjuvant is approved to mitigate these toxic manifestations (Mahipal & Pawar, 2017). Henceforth, in the present study, we plan to elucidate the mechanism of CP-induced nephrotoxicity and explore nerolidol’s protective potency using *in vitro* and *in vivo* models. Interestingly this is the first report of nerolidol on cyclophosphamide-induced nephrotoxicity. Additionally, we have shown renal fibrosis in the kidney and also reported mitigation of fibrotic mechanism by nerolidol. Not only this, but we also assess the kidney injury molecule-1, MT stain, PAS stain, and JMS stain using nerolidol in CP-intoxicated renal toxicity. Thus, the present study provides insight into the mechanism of inflammation, apoptosis, and fibrosis and the mitigation of these attributes using nerolidol.

Previously published studies have shown acute and chronic renal injury upon cyclophosphamide administration (Yung et al., 2015; Temel et al., 2020). Its administration has been reported to cause direct damage to the kidney where degeneration of the glomerulus, necrosis in the proximal convoluted tubule, distal tubules, pyknosis, etc (El-Shabrawy et al., 2020). Based on various exploratory studies, it was found that persistent oxidative stress excreted by cyclophosphamide is the hallmark of nephrotoxicity. Apart from oxidative stress, increased pro-inflammatory cytokines, apoptotic, and fibrotic protein production also play a critical role in nephrotoxicity (El-Shabrawy et al., 2020).

Moreover, it is well known that ROS and RNS are continuously produced in normal physiological conditions in response to exposure to various toxicants. Among various organs affected by these intoxicants, the kidney is mostly affected because of its checkpoint functionality (Salama et al., 2022). However, in the physiological condition, ROS and RNS are neutralized by endogenous antioxidant enzymes such as SOD, CAT, and an antioxidant such as GSH, etc. Considering the specific function of GSH, this antioxidant scavenges free radicals, and directly reverse their increased level toward normal (Salama et al., 2022). SOD, an important antioxidant enzyme, causes the conversion of superoxide free radicals into H_2_O_2_ that, when acted by the CAT, gets converted into H_2_O and O_2_ (Mahipal & Pawar, 2017). MDA is, again, an end product of lipid peroxidation of renal tissue, and its level increases during oxidative stress. On the one hand, an increased level of MDA signifies the extent of oxidative stress, whereas its increased level aggravates the inflammatory cascade (Mahipal & Pawar, 2017). Thus, an accurate balance between produced ROS, RNS, MDA, and endogenous antioxidants is critical for a healthy renal system (Ayhanci et al., 2010). Abraham & Isaac (2011) showed increased lipid peroxidation and renal damage upon CP administration as the use of glutamine significantly reduced the renal damage via its antioxidant effect (Abraham & Isaac, 2011). Similarly, Jiang et al. (2020), and Alshahrani et al. (2022) and Ghareeb et al. (2019) have also reported the significant oxidative stress caused by CP exposure and its mitigation when different natural products were used (Ghareeb et al., 2019; Jiang et al., 2020; Alshahrani et al., 2022).

It is also well-known that mitochondria are one of the essential organelles of eukaryotic cells and are accountable for various metabolic processes such as glycolysis, oxidative phosphorylation, Kerb’s cycle, and fatty acid oxidation, which ultimately leads to ATP production (Su et al., 2023). Moreover, ROS are also produced during this process at the inner mitochondrial membrane and from complexes I and III, which are also the site electron transport chain system. However, in normal physiological conditions, equilibrium is maintained between the amount of ROS produced and their scavenging via the antioxidant system (Suthar et al., 2023). According to a study published by Li et al. (2019), it was reported that renal injury is positively correlated to increased oxidative stress and apoptosis, and the profound role of Nrf2 and peroxisome proliferator-activated receptor-g coactivator-1α (PGC-1α) is noteworthy (Li et al., 2019). Alteration in their function or level to alteration in mitochondrial dynamics hamper the optimum energy production and increases ROS production. It was further found that the use of mercuric chloride in mice causes significant damage to mitochondrial dynamics, as evident by structural and ultrastructural damage. These damages eventually resulted in increased dynamin-related protein expression, reduced oxidative phosphorylation, reduced Nrf2-HO-1 level, reduced ATP production, and increased ROS level (Li et al., 2019). Similarly, Han et al. (2022) also reported that exposure to chromium causes cardiotoxic manifestation via increased oxidative stress, increased level of MDA and SOD, reduced antioxidant activity of GSH, apoptosis, altered mitochondrial dynamics, and structural damage to cardiomyocytes. In one of the published reports, when the human embryonic kidney epithelial cell line (HEK-293 T) was exposed to Hgcl_2_, silent information regulator two ortholog 1 (Sirt1) and PGC-1α levels were downregulated along with increased mitochondrial fission or mitochondrial fragmentation leading to damaged renal mitochondrial homeostasis and oxidative stress (Han et al., 2022).

Therefore, based on the above discussion, it could be inferred that using various toxic substances causes renal toxicity via persistent oxidative stress. It reduced the Nrf2-HO-1 axis, altered mitochondrial dynamics, reduced ATP production, and increased apoptosis, manifesting in structural and ultrastructural damage leading to compromised renal functions.

Now it is also important to highlight the deleterious effect of CP on mitochondrial dynamics and the protective effect of nerolidol on this axis. Ijaz et al. (2022) showed significant renal toxicity in rats when exposed to CP (Ijaz et al., 2022). Exposure to CP reduces Kerb’s cycle enzymatic activity, such as isocitrate dehydrogenase, succinate dehydrogenase, malate dehydrogenase, etc., and reduces renal mitochondrial chain complex I-IV and repolarized mitochondrial membrane potential (Ijaz et al., 2022) Also, altered mitochondrial dynamics eventually lead to significant oxidative stress, inflammation, apoptosis, and compromised renal function (Ijaz et al., 2022). Abraham & Isaac (2011) showed that exposure to CP in rats causes significant ultrastructure damage in the renal mitochondria when analyzed via TEM. The findings were similar to those of Li et al. (2019) (Abraham & Isaac, 2011; Li et al., 2019). Attia et al. (2023) also showed significant damage to cardiac mitochondria, increased MDA, and reduced antioxidant enzymes when treated with CP (Attia et al., 2023). Recently it was also showed that significant ultrastructural damage occurs to ovarian mitochondria when treated with CP. There was reduced ATP, altered MMP, swollen and vacuolated mitochondria, reduced PGC1α and SOD2 expression, and increased oxidative stress (Chen et al., 2022; Attia et al., 2023). Balakrishnan et al. (2022) showed the anticancer effect of nerolidol via modulation of mitochondrial ROS and MMP (Balakrishnan et al., 2022). Javed et al. (2016) showed that rotenone, when exposed to rodents, causes inhibition of mitochondrial complex-I leading to oxidative stress, reduced ATP level, inflammation, and other pathological attributes (Javed et al., 2016). Nerolidol, when used, effectively mitigated rotenone-induced neurotoxicity via modulation of mitochondria complex-I-related activity (Javed et al., 2016). Recently, Iqubal et al. (2019) also showed the cardioprotective effect of nerolidol against cyclophosphamide-induced cardiotoxic manifestation via reversing cardiac mitochondrial damage toward normal (Iqubal et al., 2019)

Moreover, in another study, Iqubal et al. (2019) showed increased expression of Nrf-2 in the hippocampus and cortex of mice brains against their reduced expression upon exposure to CP. The same study also found that Nerolidol binds effectively to the catalytic domain of Nrf2, eventually increasing its nuclear translocation and mitigating oxidative stress and other pathological attributes (Iqubal et al., 2019). Ceole et al. (2017) also showed that nerolidol exhibited anti-*Leishmania braziliensis* activity by targeting their ultrastructural component, such as mitochondria, and causes mitochondrial shrinkage, reduces MMP, and increases DNA degradation (Ceole et al., 2017). Therefore, based on the above discussion, it could be inferred that CP induces marked oxidative stress and may cause mitochondrial damage and altered mitochondrial dynamics. Thus, the level of ROS production and other parameters related to mitochondrial dynamics should be explored (Balakrishnan et al., 2022). Considering the effect of nerolidol on mitochondrial ROS production, and other attributes such as MMP and based on previous *in vivo, in vitro,* and *in silico* studies, it can be concluded that nerolidol effectively reversed the biochemical and structural damage to mitochondria toward normal and exhibited significant antioxidant effect.

Henceforth, looking into these facts, we, in the present study, evaluate the renal protective effect of nerolidol against cyclophosphamide-induced renal toxicity. Based on the outcome of our study, we found that exposure to CP significantly increased the level of MDA and reduced the antioxidant activity of SOD, CAT, and level GSH. Upon treatment with NERO 400 and NERO 200, NERO 400 significantly increased the antioxidant activity of SOD, GSH level, and reduced the level of MDA toward normal as shown in Figure 2. In contrast, recently, Arunachalam et al. (2021) also showed a potent antioxidant effect of nerolidol against Doxorubicin-induced cardiotoxicity. In contrast, Ni et al. (2019) and Raj et al. (2020) showed modulation of the Nrf2/HO pathway leading to an antioxidant effect. Our study’s outcome agrees with the previously published reports (Ni et al., 2019; Raj et al., 2020; Arunachalam et al., 2021).

Apart from the oxidative stress exhibited by CP, marked inflammation caused by this drug is also well-established in preclinical and clinical studies (Zhang et al., 2021). It is well-known that NF-κB is one of the critical regulators of inflammatory pathways. Various factors and stimuli regulate inflammatory attributes of NF-κB, but oxidative stress plays a potential and decisive role among those factors. There exists a close mechanistic relationship between oxidative stress and NF-κB mediated inflammation. H_2_O_2_ is one of the ROS produced during oxidative stress, and its level has been reported to cause NF-κB activation in human T cells, and upon treatment with an antioxidant, the activation was blocked (Schreck et al., 1991). NADPH oxidase NOX2 or gp91 phox is one of the key sources of ROS. It remains inactivated during the normal physiological condition but becomes active during respiratory bursts and regulates ROS production. NF-κB has been reported to activate the NADPH oxidase, eventually leading to oxidative stress (Anrather et al., 2006). Thioredoxin is one of the important and extensively studied molecules during oxidative stress and has been reported to exhibit cytoprotective effects during oxidative stress. It possesses strong free radical scavenging properties and protects cells against cytotoxic chemicals and biomolecules. Thioredoxin has been reported to inhibit the I-κB degradation that eventually leads to the mitigation of NF-κB activation (Holmgren, 2000). 8-kDa dynein light chain (LC8). In normal physiological condition, it is bound to IκBα and prevent its phosphorylation by IKK. However, in response to increased oxidative stress, LC8 oxidizes and dissociates from IκBα, leading to the phosphorylation of NF-κB and its subsequent activation (Jung et al., 2008).

Additionally, NF-κB has been reported to regulate the nitric oxide synthetase that produces reactive nitrogen species. The produced RNS reacts with the superoxide and forms reactive peroxynitrite that further reacts with the CO_2_ and forms nitrosoperoxycarbonate (ONOOCO2-), which eventually exhibits oxidative stress (Pan et al., 2012). Apart from the aforementioned mechanistic link between NF-κB and oxidative stress, the role of cyclooxygenase-2 (COX-2) is also noteworthy. In response to NF-κB activation, COX-2 becomes activated and converts the arachidonic acid to prostaglandin H2, producing ROS (Arakawa et al., 1995). Thus, NF-κB also causes oxidative stress via the COX-2 pathway. Another interaction of NF-κB and ROS has been reported via cAMP-dependent protein kinases (PKAc). Ser-276 of RelA is critical for the expression of NF-κB genes and for the interaction of RelA with CBP/300. Oxidative stress has been reported to cause phosphorylation of Ser-276 via PKAc (Zhong et al., 2002). This mechanism was further validated from the study where the use of antioxidants significantly reduced the phosphorylation of Ser-276, leading to reduced NF-κB mediated inflammatory cascade (Zhong et al., 2002). Nrf2 is an explicitly studied transcription factor concerning oxidative stress and inflammation (Li et al., 2021). A complex interplay between Nrf2 and NF-κB mediated inflammation has been reported. Reduced activity or level of Nrf2 has been reported to increase the NF-κB activity and surplus production of proinflammatory cytokines (Pan et al., 2012). Additionally, in the model of Nrf2 knock-out cells, increased activity of IKK and activation of NF-κB have been reported (Pan et al., 2012). Moreover, NF-κB has been reported to inhibit the Nrf2 via modulation of CBP-300 complex (Pan et al., 2012). Hence, reduced Nrf2 activity increases the NF-κB activity, whereas increased NF-κB reduces the Nrf2 activity.

In brief, NF-κB and ROS interact in multiple ways. Increased ROS level or reduced Nrf2 activity, on the one hand, causes NF-κB activation via modulation IκBα and IKK activity, whereas NF-κB, on the other hand, increases the level of ROS via modulation of COX-2 and other pathways.

Considering the present study, we also found that CP causes renal inflammation via inducing oxidative stress and modulation of the NF-κB signaling pathway (Temel et al., 2020). That means, upon CP administration, CP cause phosphorylation of iκB, which is attached to NF-κB (Iqubal et al., 2018). Upon phosphorylation, it undergoes nuclear translocation where nuclear translocated NF-κB stimulates the production of pro-inflammatory cytokines such as IL-6, TNF-α, IL-1β, etc., and causes renal inflammation. NF-κB is a diverse transcription factor that, apart from induction of inflammation upon stimulation by CP and ROS, also stimulated the apoptotic pathways (Khan et al., 2020). Recently, Jiang et al. (2020), Alshahrani et al. (2022), and Bokhary et al. (2022); etc., have also shown increased expression of along with an increase in the level of TNF-α, IL-6, and IL-1β leading to renal inflammation and renal damage upon cyclophosphamide administration whereas these attributes were mitigated, when natural products such as sesamin, *Cyclina sinensis*, *Salvadora persica,* etc., were used (Jiang et al., 2020; Alshahrani et al., 2022; Bokhary et al., 2022). Thus, looking into these facts, we performed *in vitro* and *in vivo* studies to ascertain the inflammation caused by CP and checked NERO's anti-inflammatory effect. In the *in vitro* study, we found a significant increase in NF-κB mRNA expression when CP was exposed to HK-2 cells. We also validated the finding of our *in vitro* study by performing immunohistochemistry of NF-κB in the *in vivo* study (renal tissue) and found a significant increase in the expression of NF-κB. As we have already discussed, causes an increased level of pro-inflammatory cytokines; hence, we also estimated their level in the renal tissue. Our finding showed an increased level of IL-6, TNF-α, IL-1β, and a reduced level of IL-10. When we tested the anti-inflammatory effect of NERO, we found that NERO, in the *in vitro* study, effectively reduced the expression of NF-κB. In the *in vivo* study also, we found a marked reduction in the expression of NF-κB along with the decrease in the level of TNF-α, IL-1β, IL-6 as well as increased in the level of IL-10 as shown in Figure 3. Henceforth, the finding of our study was in agreement with the previously published report of Ni et al. (2019), Javed et al. (2016), Fonsêca et al. (2016), Raj et al. (2020) and Akhter et al. (2022) showed that the potent anti-inflammatory effect of nerolidol against various inflammatory preclinical models (Fonsêca et al., 2016; Ni et al., 2019; Raj et al., 2020; Trindade et al., 2020).

Apoptosis, also known as programmed cell death, is a natural process by which cells are eliminated in a controlled manner in response to various physiological and pathological stimuli. Oxidative stress refers to the imbalance between ROS production and the body’s ability to detoxify them (Chiang et al., 2022). There is a strong relationship between apoptosis and oxidative stress. The relationship between oxidative stress and apoptosis is complex and involves multiple mechanisms, including signaling pathways, cellular organelles, and molecular targets. High levels of ROS can induce apoptosis by causing damage to cellular components such as lipids, proteins, and DNA, leading to cellular dysfunction and death. On the other hand, apoptosis can also generate ROS by activating specific enzymes such as NADPH oxidase and the mitochondria. Moreover, oxidative stress can also affect the signaling pathways involved in apoptosis, leading to altered gene expression and impaired cellular function. For example, oxidative stress can activate the c-Jun N-terminal kinases (JNKs) and p38 MAPK signaling pathways, which can promote apoptosis by activating pro-apoptotic proteins or inhibiting anti-apoptotic proteins (Arunachalam et al., 2021).

Oxidative stress can cause DNA damage, leading to the activation of the DNA damage response pathway, which can induce apoptosis if the damage is irreparable (El Kiki et al., 2020). Additionally, oxidative stress can overwhelm the cellular antioxidant defence systems, such as GSH and SOD, leading to increased ROS levels and subsequent apoptosis. The detailed mechanistic study further showed that increased ROS and RNS cause a discrete release of Ca^2+^ from the endoplasmic reticulum that manifests the mitochondrial membrane damage, ultimately causing cytochrome C release and increasing the level of caspase-3. Considering the mechanism of crosstalk between oxidative stress and apoptosis, it is well known that mitochondria are the primary site of ROS production (Han et al., 2019). Damaged mitochondrial DNA impedes the transcription of mitochondrial RNA, leading to disrupted electron transport chain function and increased ROS production. The increased ROS causes osmotic swelling of the matrix of mitochondria and increased permeabilization of the outer mitochondrial membrane, loss of mitochondrial membrane permeability (MMP), leading to the release of proapoptotic proteins such as cytochrome c, apoptosis-inducing factor (AIF), endonuclease G (endoG) etc. This proapoptotic protein’s release in response to oxidative stress leads to apoptosis via caspase-dependent and caspase-independent pathways (Redza-Dutordoir & Averill-Bates, 2016). In caspase-dependent apoptosis, cytochrome c is released into the cytosol, forming apoptosome in association with AIF and procaspase-9 that subsequently activate caspase 3 and form cleave caspase-3, leading to the execution of apoptosis (Redza-Dutordoir & Averill-Bates, 2016). Additionally, ROS have been reported to cause cardiolipin oxidation, leading to excess release of cytochrome C into the cytosol via the outer mitochondrial membrane (95). It stimulates the opening of mitochondrial permeability transition pores (MPTP) and membrane hyperpolarization leading to loss of MMP and cytosolic translocation of cytochrome C, Bax Bad that further leads to apoptosis (Redza-Dutordoir & Averill-Bates, 2016).

There also occurs a cross-talk between ROS and apoptosis via the p53 pathway. In normal physiological condition level of p53 is regulated by Mdm2 and via proteasomal degradation. However, during oxidative stress, p53 gets phosphorylated at Ser15 and Ser46, released from the Mdm2, bypasses proteasomal degradation, and downregulates transcription of anti-apoptotic proteins such as Bcl-2, Bcl-XL, surviving etc., and increases the transcription of proapoptotic proteins Bax, Bid etc. (Redza-Dutordoir & Averill-Bates, 2016). Additionally, p53 also undergoes mitochondrial translocation, causes mitochondrial outer membrane permeabilization, and eventually causes the release of cytochrome c into the cytosol leading to apoptosis (Redza-Dutordoir & Averill-Bates, 2016). Interestingly, antioxidant defense system such as GSH has been reported to exhibit anti-apoptotic effect via interaction with caspase-3. GSH causes S-glutathionylation at the catalytic site of caspase-3, inhibiting its proteolytic cleavage and hence inhibiting apoptosis (Redza-Dutordoir & Averill-Bates, 2016). However, during oxidative stress, the level of GSH gets reduced, and eventually, caspase-3 undergoes proteolytic cleavage leading to the production of leaved caspase 3 that causes apoptosis (Redza-Dutordoir & Averill-Bates, 2016). Studies have also shown that IRE1a in the ER gets activated in response to increased oxidative stress, which subsequently activates ASK and p38 MAPK (Ansari et al., 2019). Activation of p38 MAPK further activate CCAAT-enhancer-binding protein homologous protein (CHOP), and CHOP activate endoplasmic reticulum oxidoreductase 1 (ERO1), leading to more production of ROS. EOR1 also causes activation of inositol 1,4,5-trisphosphate (IP3R) and stimulates the release of Ca^2+^ from ER to the mitochondria via mitochondria-associated membranes, leading to mitochondrial overload, the opening of MPTP, reduced mitochondrial permeability, the release of cytochrome C and apoptosis (Redza-Dutordoir & Averill-Bates, 2016).

In brief, increased oxidative stress causes depolarization of the mitochondrial membrane, alters the permeabilization of the outer mitochondrial membrane, induces ER stress leading to increased mitochondrial Ca^2+^ overload, oxidizes cardiolipin, causes opening of MPTP, leading to the release of proapoptotic proteins, cytochrome C into the cytosol. Cytochrome C forms apoptosome complex leading to activation of caspase-3 that further undergoes proteolytic cleavage and forms cleaved caspase 3 that ultimately executes apoptosis. Increased oxidative also reduces the level of GSH that otherwise sequesters the proteolytic cleavage of caspase-3 to form cleaved caspase-3. Moreover, increased oxidative stress has been reported to modulate the p53 and JNK pathway leading to the activation of proapoptotic proteins and execution of apoptosis (Redza-Dutordoir & Averill-Bates, 2016).

Since we have already shown the oxidative stress and inflammation caused by CP and the mitigation of these attributes by nerolidol. It is also important to highlight that CP administration causes caspase-3 and cleaved caspase-3 increment and associated apoptotic factors. Considering the mechanism of renal apoptosis, as discussed above, it was found that persistent rise in ROS, reduction in antioxidant enzymes, reduced expression of Nrf2, along with increased NF-κB expression causes apoptosis in renal tissue (Yung et al., 2015; Temel et al., 2020). Sharma et al. (2017), El-Shabrawy et al. (2020), Jiang et al. (2020), Alshahrani et al. (2022), and Ijaz et al. (2022), have also shown increased renal apoptosis when exposed to cyclophosphamide, whereas treatment with natural products such as Sesamin, Picrorhiza Kurroa, Herbactin mitigated the renal apoptosis via modulation of caspase 3 levels (Sharma et al., 2017; Jiang et al., 2020; El-Shabrawy et al., 2020; Alshahrani et al., 2022; Ijaz et al., 2022). Considering these facts, we also estimated the effect of CP on the expression of caspase-3 using *in vitro* and *in vivo* studies. The findings of *in vitro* study showed increased expression of caspase-3 in the HK-2 cells when exposed to CP. In the *in vivo* study, the outcome of immunohistochemical analysis also showed increased expression of cleaved caspase-3). When we tested the anti-apoptotic effect of NERO, we found that in the *in vitro* study, NERO effectively reduced the expression of caspase-3. In the *in vivo* study also, we found a marked reduction in the expression of cleaved caspase-3 as shown in Figure 4

Increased oxidative stress and inflammation are important indicators of renal toxicity, but these are not renal-specific markers of renal injury. Hence, it is important to evaluate the effect of CP 200 on renal-specific injury markers to validate the renal injury. For the preclinical and clinical assessment of renal toxicity and the evaluation of the renal protective effect of any drug, assessment of serum urea, uric acid (UA), serum creatinine (Cr), blood urea nitrogen (BUN), etc., provide reliable information for the extent of renal damage (Mahipal & Pawar, 2017; Zhang et al., 2021). UA is a well-known end product of oxidation of the purine metabolic pathway and in normal physiological conditions, gets easily excreted (Giordano et al., 2015). An increased UA level thus signifies the altered glomerular filtration rate (GFR). Studies have also shown that increased serum UA also directly causes nephrotoxicity. It was also found that increased UA causes NADPH-dependent oxidative stress in the kidney tissue, leading to apoptosis (Giordano et al., 2015). Interestingly, it was found that UA also alters the function of fructokinase expression, leading to increased fructose activity, increased fat storage, and stimulation of monocyte chemotactic protein-1 and macrophagic activity in renal cells leading to nephrotoxic manifestations (Giordano et al., 2015).

Besides the UA, urea, BUN, and Cr are nitrogenous waste products that are also important and clinically relevant biomarkers for renal injury (Al-Naimi et al., 2019). Urea and BUN are the metabolic end product of protein metabolism. In contrast, Cr is a by-product of muscle-creatinine metabolism, and their optimum level signifies the normal functioning of the renal system (Al-Naimi et al., 2019). Apart from the importance of the aforementioned renal injury markers, kidney injury molecule-1 (KIM-1) is an extensively studied marker of renal injury (Al-Naimi et al., 2019). Interestingly, the USFDA and the European Medicine Agency for preclinical nephrotoxicity also endorsed the KIM-1 as a validated marker of renal injury (Al-Naimi et al., 2019). KIM-1 is a type1 of membrane protein and phosphatidylserine receptor that helps in the reorganization of apoptotic cells and directs them toward lysosomal degradation. Studies have shown a significant increase in the expression of KIM-1 within 48 h. of renal injury (Al-Naimi et al., 2019). Increased KIM-1 is also positively correlated with proteinuria in diabetes and related to inflammatory, fibrotic markers, and histopathological damage in various renal diseases (Bonventre, 2009). Thus, it can be concluded that increased level of UA, urea, BUN, Cr, and BUN/Cr ratio is directly related to the normal functioning of the kidney and GFR. Thus, increased urea, BUN, and Cr indicate impaired renal function, reduced GFR, and nephrotoxic manifestations (Salazar, 2014). Various published reports such as Rehman et al. (2012), Liu et al. (2016), Goudarzi et al. (2017), Sharma et al. (2017), Ghareeb et al. (2019), Ebokaiwe et al. (2021), Waz et al. (2021), Hu et al. (2022), Ijaz et al. (2022), etc., have shown increased serum level of kidney injury markers such as creatinine, uric acid, blood urea nitrogen, and serum urea along with increased KIM-1 level when exposed to cyclophosphamide (Rehman et al., 2012; Goudarzi et al., 2017; Sharma et al., 2017; Ghareeb et al., 2019; Waz et al., 2021; Hu et al., 2022; Ijaz et al., 2022).Thus, in the present study, we have estimated the serum level of these renal injury markers, calculated the BUN/Cr ratio, and evaluated the expression level of KIM-1 to strengthen and validate the findings. We found that CP 200 administration significantly increased the serum level of UA, urea, BUN, Cr, and BUN/Cr ratio, along with the expression of KIM-1 whereas treatment with NERO 400 significantly reversed their derailed level toward normal and hence signifies the nephroprotective effect as shown in Figure 5.

A major challenge for the administration of CP is the damage to the structural integrity of renal tissue that eventually manifests into other renal toxic manifestations such as altered GFR rate, disturbance in overall filtration mechanism, and altered pH balance (Abraham & Isaac, 2011). Besides the direct effect of CP on renal histopathological damage, persistent increases in oxidative stress, inflammation, and apoptosis also participate in renal damage (Temel et al., 2020). Thus, in the present study, we also evaluated the extent of renal tissue damage upon CP 200 administration. The outcome of our study showed significant glomerular degeneration, distorted basement membrane, pyknosis, damaged mesangial and endothelial cells, cellular disintegration, and damaged (proximal convoluted tubules and distal convoluted tubules. In addition to our findings, various published reports such as Rehman et al. (2012), Liu et al. (2016), Goudarzi et al. (2017), Sharma et al. (2017), Ghareeb et al. (2019), Ebokaiwe et al. (2021), Waz et al. (2021), Hu et al. (2022), and Ijaz et al. (2022) etc., have shown increased histopathological aberrations such as pyknosis, dilation of Bowmen’s capsule, leukocytic infiltration, thicken Bowman’s capsule, disrupted basal laminae etc. when exposed to cyclophosphamide (Rehman et al., 2012; Goudarzi et al., 2017; Sharma et al., 2017; Ghareeb et al., 2019; Waz et al., 2021; Hu et al., 2022; Ijaz et al., 2022). However, when NERO 200 and NERO 400 were used as an adjuvant, the outcome of the study showed a significant reversal to aforementioned renal histopathological aberrations toward normal with NERO 400 and the findings of our study agreed with previously published reports, as mentioned above and as shown in Figure 6.

To further validate the finding of the histopathological study, we performed the Jones Methenamine Silver Stain (JMS). JMS is a specific stain for analyzing the basement membrane of the glomerulus and mesenchymal cells in the kidney (Herrera & Lott, 2013). JMS staining helps to visualize thickened or damaged basement membranes and is often seen in diabetic kidneys (Nicholas & Jacques, 2005; Herrera & Lott, 2013). Interestingly, in the H&E staining, these thickened or damaged basement membranes are not distinguishable. It is also important to highlight that the thick basement membrane signifies membranous nephropathy (Nicholas Cossey et al., 2020). The basement membrane is an integral part of the glomerulus and supports the filtration of waste material and other extra fluids from the blood. Studies also signify that a thick basement membrane is among the most common cause of nephrotic syndrome in which altered flow of urine, reduced protein level, hyperchloremia, etc., are seen (Miner, 2012; Nicholas Cossey et al., 2020). In the present study, we have not focused on nephrotic syndrome. Instead, we tried to explore the possible nephrotic manifestations induced by CP uses. Thus, more focused studies are needed to rule out membranous nephropathy or nephrotic syndrome. The present study reveals the thickened and damaged glomerular and mesenchymal basement membrane upon CP 200 administration when compared with control groups (*P* < 0.001). When NERO 400 was used as an adjuvant, a significant reveal of these manifestations toward normal was found (*P* < 0.001), whereas NERO 200 was found to be ineffective (*P* > 0.05), as shown in Figure 7.

As discussed above, we showed the thickening of the basement membrane upon CP administration. Herein we also tried to assess the effect of CP 200 on renal glycogen storage and associated complications. PAS staining is used to validate the thickening of the basement membrane as JMS does along with the dilation of Bowman’s space. Particularly in the kidney, this staining distinguishes tubulitis or diabetic Kimmelstoel-Wilson nodules, which stain PAS positive (van Raaij et al., 2018). Amyloidosis stain PAS negative. In the present study, we found a higher intensity of PAS staining in the glomerulus and interstitials. The extent of this deposition somehow correlated with the etiology of glycogen storage disease (GSD) (van Raaij et al., 2018). A common connecting link between GSD renal dysfunction was found to be hyperuricemia, increased uric acid, and an increase in other markers of renal injury (Al Drees et al., 2017).

Interestingly, in the present study, we also found an increase in these markers of renal injury. However, we are not confirming the presence of GSD in the kidney but highlighted that CP administration increases glycogen deposition in the kidney, which might be responsible for nephrotoxic manifestations. Previously, Al-Gayyar et al. (2016), El-Shabrawy et al. (2020), Sharma et al. (2017), Sheth et al. (2018), and Jiang et al. (2020), etc. have explored the effect of cyclophosphamide upon PAS staining and found that CP treated group exhibit Bowman’s capsular dilation, damaged or thicken basal laminae, deformed glomeruli, increase in the capsular space of (Al-Gayyar et al., 2016; Sheth et al., 2018; El-Shabrawy et al., 2020; Jiang et al., 2020; Sharma et al., 2021). The present study’s finding showed the thickening of the glomerular and mesenchymal basement membrane and glycogen deposition in the glomerulus and the interstitials upon CP 200 administration. When NERO 400 was used as an adjuvant, a significant reversal of these manifestations toward normal was found and the findings of our study were in agreement with previously published reports, as mentioned above and shown in Figure 8.

As we have already seen and discussed the effect of cyclophosphamide on oxidative stress, inflammation, apoptosis, and histopathological damage, it is also important to understand that apart from these attributes, CP-induced fibrosis is a limiting factor for its use (Grynberg et al., 2017). In the etiology of renal fibrosis, tubulointerstitial fibrosis, and glomerulosclerosis are the hallmark pathological feature and often lead to chronic kidney disease (CKD) (Yung et al., 2015). CKD is considered one of the challenging conditions in nephrology and often causes end-stage renal disease, where dialysis and renal transplant remains the only option. Fibrosis in renal tissue is characterized by excess extracellular matrix (ECM) deposition and abnormal activation of multiple signaling pathways (Wu et al., 2013). Considering molecular pathogenesis of renal fibrosis, activated epithelial-mesenchymal transition (EMT), infiltration of macrophage or monocytes, inflammatory cytokines infiltration, and apoptosis are pivotal ones (Nogueira et al., 2017). Additionally, studies have shown that, upon persistent renal-toxic stimulus, osteopontin, vascular cell adhesion molecules (VCAMs), intracellular adhesion molecules (ICAMs) also get activated and move toward the interstitials as well as into the glomerulus and participate in the fibrotic cascade via production of inflammatory and fibrogenic molecules (Humphreys, 2018). TGF-β is one of the well-explored and we-established pro-fibrotic markers in fibrotic disorders, including renal fibrosis (Sharma et al., 2017). It is produced by infiltrating leukocytes as well as by renal cells. TGF-β exists in the three isoforms, and all these isoforms are involved in renal fibrosis via the modulation of Smad signaling pathways (Hu et al., 2018). TGF-β exists in three isoforms, namely TGF-β1, TGF-β2, and TGF-β3, among which TGF-β1 is the common most isoform. Initially, TGF-β1 is released in association with LAP (latency-associated peptide), remains inactive, and LTBP (latent TGF-β-binding protein) (Hu et al., 2018). Upon exposure to the toxic stimulus or increased ROS, it becomes active, becomes free from LAP and LTBP, and binds with TGF-β1 receptors. Upon binding, receptors-associated Smads (i.e., Smad 1, Smad2, and Smad3) become phosphorylated and undergo nuclear translocation leading to the transcription of pro-fibrotic genes. Besides the Smad-dependent fibrotic pathway, TGF-β also causes renal fibrosis via modulation of p38 MAPK, Rho-GTPase, and NF-κB pathway (Meng et al., 2013). Considering the mechanism of TGF-β1 in renal fibrosis, it was found that TGF-β1 causes increased production of ECM like collagen I and fibronectin via Smad3-dependent pathways such as induction of tissue inhibitor of metalloproteinase and trans differentiation of myofibroblast, epithelial cells (Wu et al., 2013). Moreover, TGF-β1 also acts directly on resident cells of renal tissue, stimulating mesangial cell proliferation and increasing the matrix’s production, leading to renal injury and fibrosis (López-Hernández & López-Novoa, 2012). Mechanistically, TGF-β causes stimulation of myofibroblast and transition of interstitial fibroblast and mesangial cells that eventually become fibrogenic cells (Wu et al., 2013). TGF-β has been reported to induce apoptosis that results in the depletion of podocytes, peritubular capillaries, glomerular loss, glomerulosclerosis, and interstitial myofibroblast generation that further leads to fibrosis (Hu et al., 2018). It is further important to understand the profibrotic role of Smad3 in renal fibrosis. We have already discussed that TGF-β1 is accountable for the phosphorylation of Smad3. Smad2 and Smad3 are almost similar (90%) but exhibit opposite effects, i.e., Smad2 is renal protective, whereas Smad3 is actively involved in renal fibrosis. Smad3 has been reported to bind directly with the collagen’s promotor region and stimulate its production and inhibit the degradation of ECM (Vindevoghel et al., 1998). Pro-fibrotic role of Smad3 in renal fibrosis was further confirmed by the study where Smad3 deletion suppressed the fibrosis in a rodent model of fibrogenesis (Zhou et al., 2010). Moreover, the use of an inhibitor of Smad3 significantly reduced the endothelial myofibroblast transition and showed renal protection (Li et al., 2010).

Apart from the aforementioned discussed mechanism of renal fibrosis, the role of -oxidation is also important to address. β-oxidation is a complex process and involves the pivotal role of fatty acid transportation in the matrix of mitochondria under the influence of palmitoyl transferase I (CPT1) (Kang et al., 2015). Apart from the role of CPT1, acyl-CoA dehydrogenase (CAD), malonyl CoA, and acyl-CoA also play important roles in the process of β-oxidation, leading to optimum production of ATP (Kang et al., 2015). Any alteration in these molecules’ functioning results in impaired β-oxidation and reduced ATP production. Reduced ATP has been reported to initiate the production of inflammatory and fibrogenic signaling molecules, leading to renal fibrosis (Kang et al., 2015). Considering the role of CP in fibrosis, we have already shown that CP causes hepatic and cardiac fibrosis. In contrast, Sayed-Ahmed et al. (2014) also reported the mRNA reduction of CPT1 and increased expression of malonyl CoA that leads to reduced β-oxidation upon CP administration (Sayed-Ahmed et al., 2014). Looking into these facts, we explored the effect of CP on the expression of TGF-β1, level of TGF-β1, level of HLA, and 4-HP and also performed the Masson’s trichrome stain in the renal tissue. Hence, it can be inferred that TGF-β1/Smad3 signaling pathways are pivotal in renal inflammation and fibrosis via advanced end products (AGEs) and C-reactive protein (CRP) stress molecules. Thus, TGF-β1/Smad3 axis regulates the multiple target genes, miRNAs, etc., leading to renal inflammation, apoptosis, and fibrosis. Henceforth, TGF-β1/Smad3 targeting appears to be a novel strategy to combat cyclophosphamide-induced renal inflammation and fibrosis. TGF-β1, Smad3, HLA, and 4-HP have among the clinically used biomarkers and are positively correlated with the severity of fibrosis. Al-Gayyar et al. (2016), Sheth et al. (2018), El-Shabrawy et al. (2020) and Jiang et al. (2020) have shown increased renal fibrosis when treated with cyclophosphamide (Sheth et al., 2018; Al-Gayyar et al., 2016; El-Shabrawy et al., 2020; Jiang et al., 2020). Al-Gayyar et al. (2016) have shown the profibrotic effect of CP via the increased level of TGF-β1 and treatment with Nigella sativa oil significantly reduced the renal fibrosis toward normal (Al-Gayyar et al., 2016). Moreover, recently, Salama et al. (2022) have shown that CP administration causes renal fibrosis via modulation of TGF-β1/Smad3 pathways, and alogliptin treatment reversed the renal fibrosis toward normal (Salama et al., 2022). Hence in the present study, we have estimated the markers of renal fibrosis. We found that CP 200 increased the expression of TGF-β1, level of TGF-β1, HLA, 4-HP (*P* < 0.001) and also increased the fibrotic area as evident from MT stain. When we used NERO 200 and NERO 200, NERO 400 showed a marked reduction in the expression of TGF-β1, level of Smad3 HLA, 4-HP (*P* < 0.01) and also reduced the collagen-rich area in the MT staining and the findings of our study were in agreement with previously published reports, as mentioned above and as shown in Figure 9.

## 4. Conclusions

The outcome of the present study showed the potent renal protective potency of nerolidol against cyclophosphamide-induced renal toxicity. We found that administration of a single dose of cyclophosphamide 200 mg/kg, p.o induced renal toxicity via oxidative stress, inflammation, apoptosis, fibrosis, and histopathological aberrations. In the *in vivo* study, when Nerolidol was used at the dose of 400 mg/kg, a potent antioxidant, anti-inflammatory, anti-apoptotic, and antifibrotic effect via increased activity and level of SOD, CAT, GSH, and reduced level of MDA was observed. Nerolidol 400 mg/kg also reduced the expression of -κB, cleaved caspase-3, and Smad3 along the level of TNF-α, IL-6, Il-1β, kidney injury markers, TGF-β1, and other fibrotic markers. In the *in vitro* study, nerolidol at the 50 µM reduced the expression of NF-κB, caspase-3, and TGF-β1. However, no significant renal protection was exhibited by the lower dose of nerolidol in the *in vivo* (200 mg/kg, p.o) as well as *in vitro* study (25 µM). This ineffectiveness could be due to lower doses and pharmacokinetic limitations such as low bioavailability and low solubility, resulting in inferior therapeutic outcomes. We further conclude that the estimation of p-Smad2/3, mitochondrial ROS, and redox signaling pathways should be explored, and it can be considered a limitation of this study. Furthermore, more detailed cellular and molecular studies are needed to use nerolidol as an adjuvant among patients treated with chemotherapeutic drugs.
